# Broad MICA/B Expression in the Small Bowel Mucosa: A Link between Cellular Stress and Celiac Disease

**DOI:** 10.1371/journal.pone.0073658

**Published:** 2013-09-13

**Authors:** Yessica L. Allegretti, Constanza Bondar, Luciana Guzman, Eduardo Cueto Rua, Nestor Chopita, Mercedes Fuertes, Norberto W. Zwirner, Fernando G. Chirdo

**Affiliations:** 1 Laboratorio de Investigación en el Sistema Inmune – LISIN, Departamento de Ciencias Biológicas, Facultad de Ciencias Exactas, Universidad Nacional de La Plata, La Plata, Argentina; 2 Servicio de Gastroenterología, Hospital de Niños “Sor María Ludovica,” La Plata, Argentina; 3 Servicio de Gastroenterología, Hospital San Martin La Plata, La Plata, Argentina; 4 Laboratorio de Fisiopatología de la Inmunidad Innata, Instituto de Biología y Medicina Experimental (IBYME), Consejo Nacional de Investigaciones Científicas y Técnicas (CONICET), Buenos Aires, Argentina; 5 Departamento de Química Biológica, Facultad de Ciencias Exactas y Naturales, Universidad de Buenos Aires, Buenos Aires, Argentina; 6 Departamento de Microbiología, Parasitología e Inmunología, Facultad de Medicina, Universidad de Buenos Aires, Buenos Aires, Argentina; Tulane University, United States of America

## Abstract

The MICA/B genes (*MHC class I chain related genes A and B*) encode for non conventional class I HLA molecules which have no role in antigen presentation. MICA/B are up-regulated by different stress conditions such as heat-shock, oxidative stress, neoplasic transformation and viral infection. Particularly, MICA/B are expressed in enterocytes where they can mediate enterocyte apoptosis when recognised by the activating NKG2D receptor present on intraepithelial lymphocytes. This mechanism was suggested to play a major pathogenic role in active celiac disease (CD). Due to the importance of MICA/B in CD pathogenesis we studied their expression in duodenal tissue from CD patients. By immunofluorescence confocal microscopy and flow cytometry we established that MICA/B was mainly intracellularly located in enterocytes. In addition, we identified MICA/B^+^ T cells in both the intraepithelial and lamina propria compartments. We also found MICA/B^+^ B cells, plasma cells and some macrophages in the lamina propria. The pattern of MICA/B staining in mucosal tissue in severe enteropathy was similar to that found in *in vitro* models of cellular stress. In such models, MICA/B were located in stress granules that are associated to the oxidative and ER stress response observed in active CD enteropathy. Our results suggest that expression of MICA/B in the intestinal mucosa of CD patients is linked to disregulation of mucosa homeostasis in which the stress response plays an active role.

## Introduction

The MICA/B genes encode proteins that are distantly related to the HLA class I gene products. They do not associate with β_2_-microglobulin and are conformationally stable without conventional MHC class I peptides bound. Thus, MICA/B molecules have no role in antigen presentation. In addition, MICA is rapidly up-regulated under different stress conditions such as heat-shock, oxidative stress, transformation and viral infection [1]–[5].

MICA/B interact with the activating NKG2D receptor which is constitutively expressed on NK cells, CD8^+^ α/β T cells, peripheral blood and intestinal intraepithelial γ/δ T cells, and NKT cells. NKG2D functions as co-stimulatory signal on T cells and as a primary recognition receptor on NK cells [6]. Upon engagement, NKG2D triggers a cytotoxic response and IFN-γ secretion. Consequently, MICA/B have been considered markers of cellular distress that facilitate the elimination of damaged, infected, or transformed cells and serving as an immune surveillance mechanism [7]–[9].

MICA has been also suggested to play a role as target molecule of the innate response in the intestinal mucosa in active Celiac Disease (CD) [10], [11]. CD is a chronic immune-mediated enteropathy developed in genetically predisposed individuals exposed to a group of proteins present in wheat, rye, barley and oats. The lesion is limited to the small intestine and characterized by a remodeling of the mucosal architecture with villous atrophy, crypt hyperplasia and lymphocyte infiltration both in lamina propria and intraepithelial compartments. The current treatment is a life-long gluten-free diet (GFD), which results in a complete remission of symptoms and recovery of normal histology. Gluten derived peptides, many of them selectively deamidated by transglutaminase 2, are presented by certain dendritic cell subsets in a HLA-DQ2/DQ8 restricted manner, while gluten specific intestinal CD4^+^ T cells characteristically produce large amounts of IFN-γ determining the well-known Th1 pattern associated to CD [12].

Mechanisms from both innate and adaptive immunity are involved in CD pathogenesis and cross-talk between them contributes to disease progression. Innate immunity contributes to the occurrence of structural changes at the intestinal mucosa. Many biological and proinflammatory effects have been described for p31–43, one of the most studied gliadin peptides, such as induction of enterocytes apoptosis and IL-15 production [13], [14].

MICA/B have a restricted expression in normal tissues and were originally described in gut epithelial cells [2]. In contrast to MHC class I genes, MICA/B promoters contain heat shock response elements that are involved in their upregulated expression observed under stress conditions [2]–[4].

IL-15, a key cytokine upregulated in intestinal mucosa in active CD [15]–[17], was shown to be involved in the induction of cell surface expression of MICA on intestinal epithelial cells and also regulates the cytotoxic activity of intraepithelial lymphocytes (IEL). Consequently, surface expression of MICA has been postulated as signal for enterocytes killing upon engagement of NKG2D on IELs in an IL-15-rich environment. Notably, increased MICA expression in active CD returned to normal under gluten-free diet, highlighting the importance of the signals derived from gliadin-derived peptides in its up-regulation [10], [11], [18], [19].

Although it has been postulated that MICA/B play a relevant role in the elimination of damaged/stressed epithelial cells and therefore in gut homeostasis, its expression in the context of the ongoing stress response in the intestine of CD patients has not been analyzed. Thus, the aim of this study was to perform an extensive analysis of the pattern of MICA/B expression in intestinal mucosa of CD patients, and study its possible link to the ongoing stress response in the mucosa. We found intracellular expression of MICA/B in enterocytes as well as in distinct populations of immune cells in both the intraepithelial and *lamina propria* compartments of the intestinal mucosa. Remarkably, MICA/B^+^ T cells were found among intraepithelial lymphocytes (IELs) and in *lamina propria*, and the number of these cells was increased in severe enteropathy. We also found that the pattern of MICA/B expression in CD enteropathy was similar to that observed in *in vitro* stress models.

## Patients and Methods

### Biopsy Specimens

Intestinal biopsies were taken from patients younger than 5 years old suffering from gastrointestinal symptoms following the routine procedure to diagnose celiac disease.

Patients were classified into five groups according to histology. 49 patients comprised the “severe enteropathy group” (all of them had celiac disease with atrophic mucosal architecture and positive anti-endomysial antibodies -EMA-), 29 patients constituted the “moderate enteropathy group” in which the villous height/crypt depth ratio was between 1 and 2, and 24 patients constituted the “mild enteropathy” group, with a villous height/crypt depth ratio of 2 to 2.5. In these last two groups, serological tests and clinical symptoms were compatible with CD. Four patients constituted the “gluten free diet group”, presenting moderate or severe enteropathy in their first biopsy diagnosis and total recovery of normal gut architecture after at least two years on a strict gluten free diet. The “control group” included 44 EMA negative patients suffering from dyspepsia (n = 22) or upper abdominal pain (n = 22); all these patients had intestinal biopsies with normal histology. For flow cytometric analysis on epithelial cells of duodenal samples and confocal analysis some adult biopsy specimens were also used. For this purpose control samples were classified as EMA negative patients suffering from dyspepsia and celiac patients belonged to the “severe enteropathy group” (with atrophic mucosal architecture and EMA positive).

The present study was performed with a written informed consent from the patient or her/his parent or legal guardian, and the approval by the Ethical Committee of the Instituto de Investigaciones Pediátricas. Hospital de Niños Sor María Ludovica from La Plata (Buenos Aires, Argentina).

### Duodenal Biopsy Specimens’ Conservation and Culture

During the upper-gastrointestinal endoscopy, five distal duodenum biopsy specimens were collected. One specimen was fixed in Bowin’s medium for histological analysis to confirm CD. The others were used for culture and/or RNA isolation. For biopsy culture, samples were incubated for 3 or 24 h at 37°C in medium alone or in medium supplemented with 50 ng/ml of human recombinant IL-15 (BD Pharmingen), 100 µg/ml p31–43 gliadin peptide (Biomedal, Spain) or IL-15 and p31–43 together in RPMI medium supplemented with penicillin 62,4 µg/ml (Bagó Laboratories), streptomycin 100 µg/ml (Bagó Laboratories), gentamicin 0,5 g/l and fetal calf serum (Gibco) 10%. After culture, samples were washed in HBSS/gentamicin 0,5 g/l and total RNA was extracted.

### Histological Classifications

Small-bowel mucosal morphology was determined under light microscopy from 8 well-oriented biopsy sections stained with hematoxylin and eosin; poorly orientated samples were not taken into consideration and were discarded from the study. Histological classification was performed for clinical purposes following reported criteria by measuring villous height/crypt depth ratio (Vh/CrD) in at least 5 well-oriented villous-crypt pair and expressed as mean ± SD.

### Antibodies

The anti-MICA/B monoclonal antibody (mAb) D7 [20] was used to assess MICA/B expression. Monoclonal mouse IgG2b (BPC4, Ancell) was used as isotype-matched negative control antibody (IC). Antibodies to CD138 (MI15, syndecan1), CD68 (P6-M1), HAM 56 (MO632), CD3 (M7254 and Polyclonal Rabbit Anti-Human, (A0452)) and CD20 (L26) were obtained from DAKO; as well as the Target Retrieval Solution **(**S1699) and DakoCytomation fluorescent mounting medium (S3023). Anti-CD7 (CD7.272) was from Novocastra; anti-CD1a (MOB363) was from Diagnostic Biosystems, anti-CD11c (EP1347Y) was from Abcam; anti-CD3-PECy5 (UCHT1) was from BD Biosciences; rabbit monoclonal anti-BIP/grp78 (C50B12) was from Cell Signaling; Cy3-labeled streptavidin and Cy5-labeled donkey anti-goat IgG (705-175-147) were from Jackson ImmunoResearch; Goat polyclonal anti-TIA-1 IgG (sc-1751) and FITC-labeled goat anti-mouse IgG (sc-2010) were from Santa Cruz Biotechnology. FITC-labeled anti-rabbit IgG (RG-96) and propidium iodide (P4170) were from Sigma; Alexa 488 goat anti-rabbit IgG (A11008) and Alexa 594 F(ab´)_2_ fragment goat anti-mouse IgG (A11020) were from Invitrogen, (USA). Counterstaining was performed using DAPI Nucleic Acid Stain and SYTO® 13 Green Fluorescent Nucleic Acid Stain (S-7575), both from Invitrogen (USA).

### Immunohistochemical Staining

Bowin’s-fixed, paraffin-embedded 5-µm–thick small bowel biopsy sections were rehydrated, blocked with normal horse serum, and stained with 15 µg/ml of the D7 mAb or the IC mAb. Bound antibodies were detected with the Vector Vectastain ABC kit and Peroxidase substrate kit (DAB, Vector Vectastain) following the instructions provided by the manufacturer. Samples were counterstained with Haematoxylin, dehydrated with alcohol, and mounted. An arbitrary score of intensities was used to compare samples. This score was defined in numbers from one to four, according to the intensity of immunoperoxidase staining; isotype control was defined as score = 0 (zero). Samples were analyzed in a Nikon Eclipse E400 microscope. Three well oriented slides per patient were used for the staining. For this and all the analysis made on tissue sections, slides were divided into units of *muscularis mucosae* (m.m.). One unit of m.m. represents an area of 6 crypts. Two to four units of m.m. were analyzed per sample. Analysis was performed blindly by two investigators. The whole study was performed twice.

### Confocal Microscopy

Intestinal biopsy samples were frozen in OCT embedding compound on dry ice and stored at −70°C. Tissue sections (6 µm) were fixed in acetone or in Bowin’s solution and included in paraffin. Bowin’s-fixed, paraffin-embedded rehidrated biopsy sections or acetone-fixed sections were blocked with inactivated normal horse serum or normal goat serum. Sections were incubated sequentially with lineage specific mAbs, followed by FITC-labeled goat anti-mouse IgG or anti-rabbit IgG. Blocking was performed with 5% inactivated normal mouse serum and sections were incubated with 150 µg/ml biotinylated mAb D7 followed by Cy3-labeled streptavidin. DAPI was used for nuclei staining. Isotype control (IC) was used in all cases. For single MICA/B staining, counterstaining was performed using SYTO® 13 Green Fluorescent Nucleic Acid Stain. For BIP staining, samples were rehidrated, blocked with inactivated normal goat serum, and incubated sequentially with anti-BIP mAb 1/50 and FITC-labeled anti-rabbit IgG. For single staining samples were then incubated with propidium iodide (1 µg/ml). For double staining, samples were further incubated with anti-CD138 mAb followed by Alexa 594 F(ab´)_2_ fragment goat anti-mouse IgG. Samples were then dehydrated through alcohol and mounted.

For stress co-localization studies, frozen or rehydrated paraffin embedded sections were blocked and incubated with Goat polyclonal IgG anti-TIA-1 Ab followed by Cy5-labeled donkey anti-goat IgG, and then with 150 µg/ml biotinylated mAb D7, followed by Cy3-labeled streptavidin. DAPI was used for nuclei staining. IC was used in all cases.

Double staining of slides with samples from patients from all groups were counted for total number of CD7^+^ cells and double positive (MICA/B^+^CD7^+^) cells, in the lamina propria and intraepithelial compartments. The same was performed for the CD138^+^ population in lamina propria. Number of double positive cells and total number of cells per population were determined blindly in each unit of m.m. analyzed of each patient. At least, two to four units were counted in each sample and the median percentage of all the units counted of one sample were plotted.

Images were acquired using either a PASCAL-LSM Confocal Laser Scanning Microscope (Carl Zeiss, Oberkochen, Germany), or a TCS SP5 Leica confocal Microscope. Images processing was preformed using the LSM 5 v 3.2 software and the Leica LAS AF software, respectively.

### Cell Culture and Stress Induction

The human colon adenocarcinoma cell line Caco-2, American Type Culture collection (ATCC), was propagated in Dulbecco’s modified Eagle’s medium (DMEM, Sigma), supplemented with 15% fetal bovine serum (FBS, (Gibco), 1% HEPES buffer solution 1 M (Gibco), 1% penicillin/streptomycin (Sigma) and 1% MEM non-essential Amino Acids (Gibco).

Three different stress models were used to study MICA/B expression in Caco-2 cells; endoplasmic reticulum stress due in response to calcium starvation was induced with 1 µM Thapsigargin (Sigma), oxidative stress was induced using 250 or 500 µM Sodium Arsenite (Sigma) and fever-range thermal stress was induced exposing the cells to 42°C for one hour. Induction of stress response was confirmed by confocal microscopy as presence of positive cytoplasmic TIA-1 stress granules. MICA/B expression under stress conditions was studied using confocal microscopy.

### Immunofluorescence Studies on Caco-2 Cells

Cells exposed to stress stimuli were washed with PBS and fixed in 4% p-formaldehyde and 4% sucrose, followed by two washes with NH_4_Cl 50 mM. Cells were then permeabilized in 0.1% Triton X-100 and blocked using 2% BSA (Sigma) for 60 min at room temperature. Cells were incubated sequentially with anti-MICA/B mAb D7 or IC antibodies, FITC-labeled goat anti-mouse IgG, and counterstained with DAPI. Double staining was assessed using goat polyclonal anti-TIA-1 IgG followed by Cy5-labeled donkey anti-goat IgG after which cells were stained with 150 µg/ml of biotinylated mAb D7, followed by Cy3-labeled streptavidin and counterstained with DAPI. IC were used in all cases.

### Isolation of Epithelial Cells and Flow Cytometric Studies

Four biopsy samples were taken from each patient, washed with calcium and magnesium free HBSS (Gibco) containing 1 mM EDTA (Sigma, USA) and incubated at 37°C for 20 minutes. Then samples were shaked vigorously to dislodge cells until a cloudy suspension was obtained. Samples were filtered through an 80-µM filter mesh (BD Biosciences, San Jose, CA, USA) in 50 ml Falcon tubes, and centrifuged at 400 g for 10 minutes 4°C. Supernatant was discarded and the cell pellet was washed. For flow cytometric analysis 0.5×10^6^ cells/tube were incubated with inactivated human serum to block Fc receptors. Surface and intra-cytoplasmic staining were analyzed. For intracellular staining cells were treated with Fixation&Permeabilization kit (eBiosciences, San Diego, USA). Cells were incubated with anti-CD3-PECy5 and/or anti MICA/B D7 mAb, followed by anti-mouse IgG-FITC. IC were used in all conditions tested. Cells were analyzed in a BD FACSCalibur™ flow cytometer (BD Bioscience) and data were processed using CELLQest™ (BD Bioscience) and FlowJo (Tree Star Inc., Ashland, OR, USA) software.

### Statistical Analysis

GraphPad Prism 4 software (GraphPad, San Diego, CA) was used for statistical analysis and plotting. Non parametric Kruskal Wallis test followed by Dunńs multiple-comparison posttest or the nonparametric Mann-Whitney U test were used to analyze data. A p value <0.05 was considered statistically significant.

## Results

### MICA/B Expression in the Small Intestine Epithelium

**Figure 1:**
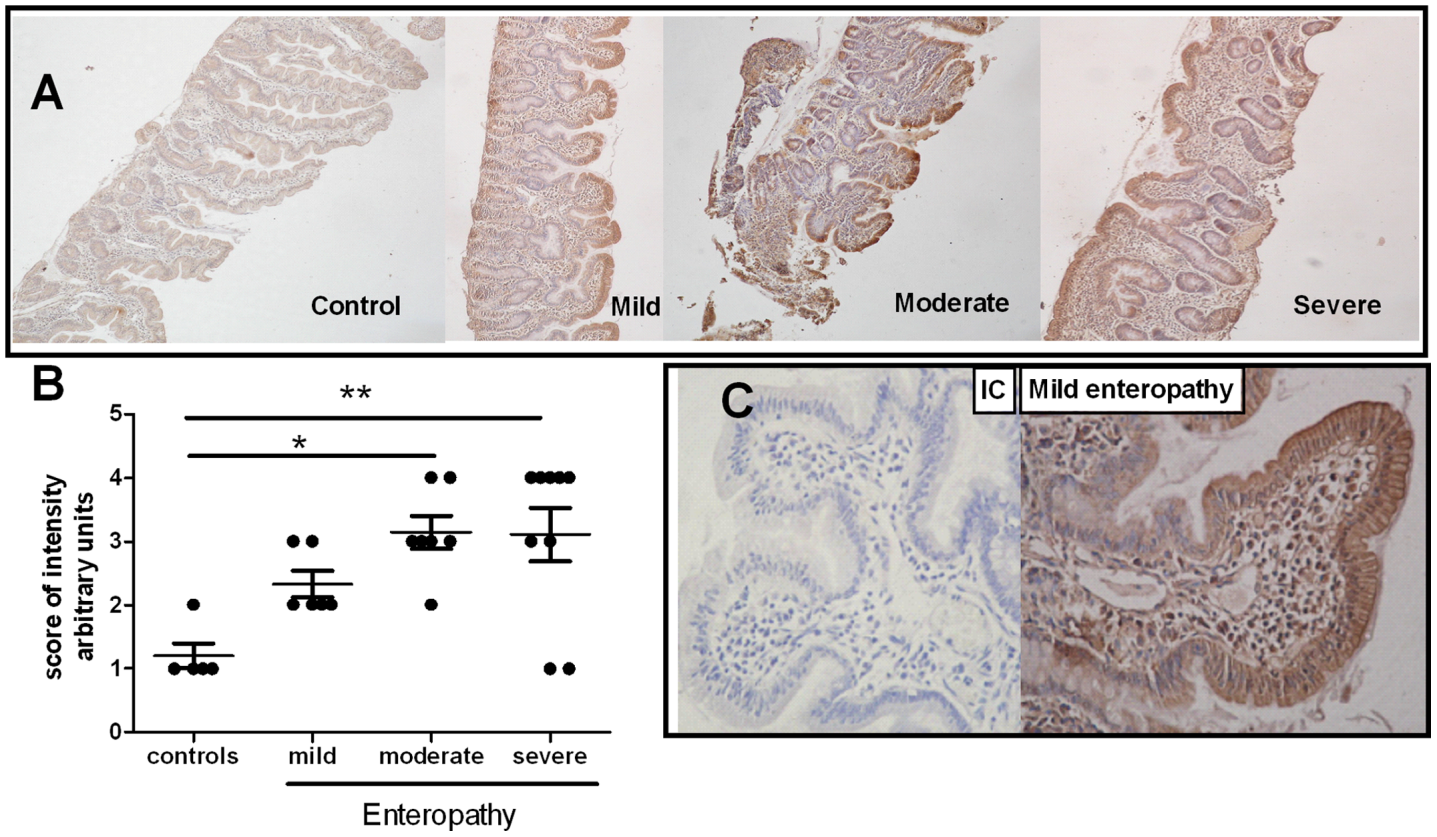
MICA expression in intestinal mucosa of CD patients. **A.**- Representative immunoperoxidase staining of MICA/B in intestinal biopsy sections from pediatric CD patients with different degrees of lesion (mild, moderate and severe enteropathy; magnification 20×). **B.**- Immunohistochemical analysis for MICA/B expression in sections of intestinal biopsies from 27 pediatric patients. An arbitrary score of intensity of staining was used (from 0 to 4). The IC control antibody was defined as score zero. Each dot corresponds to the score obtained for each sample. * *p*≤0,05; ** *p*≤0,01 (Non parametric Kruskal wallis test followed by the Dunns multiple-comparison posttest).**C.**- Pattern of MICA/B expression along the epithelium on a mild enteropathy sample. Isotype Control (IC) is shown (magnification 40×).

Immunohistochemical analysis to detect MICA/B expression revealed that the epithelium of duodenal samples from CD patients had increased MICA/B expression compared to that of healthy controls (**Figure 1A**). When samples were grouped according to the histological evaluation into non celiacs with normal architecture, and celiacs with mild, moderate or severe enteropathy (villous atrophy and crypt hyperplasia), we observed that intestinal mucosa from untreated CD patients exhibited a higher intensity of MICA/B staining compared to samples from healthy controls. There was also a positive trend between the degree of lesion and the intensity of the staining **(** **Figure 1B** **)**. Most of the samples with enteropathy showed a discontinuous pattern of MICA/B expression along the epithelium. In all cases analysed, enterocytes from the top of the villi were the most intensively stained cells in the epithelia (**Figure 1C**). Remarkably, in addition to enterocytes, other cells, such as intraepithelial lymphocytes and mononuclear cells in lamina propria, also exhibited MICA/B expression.

**Figure 2:**
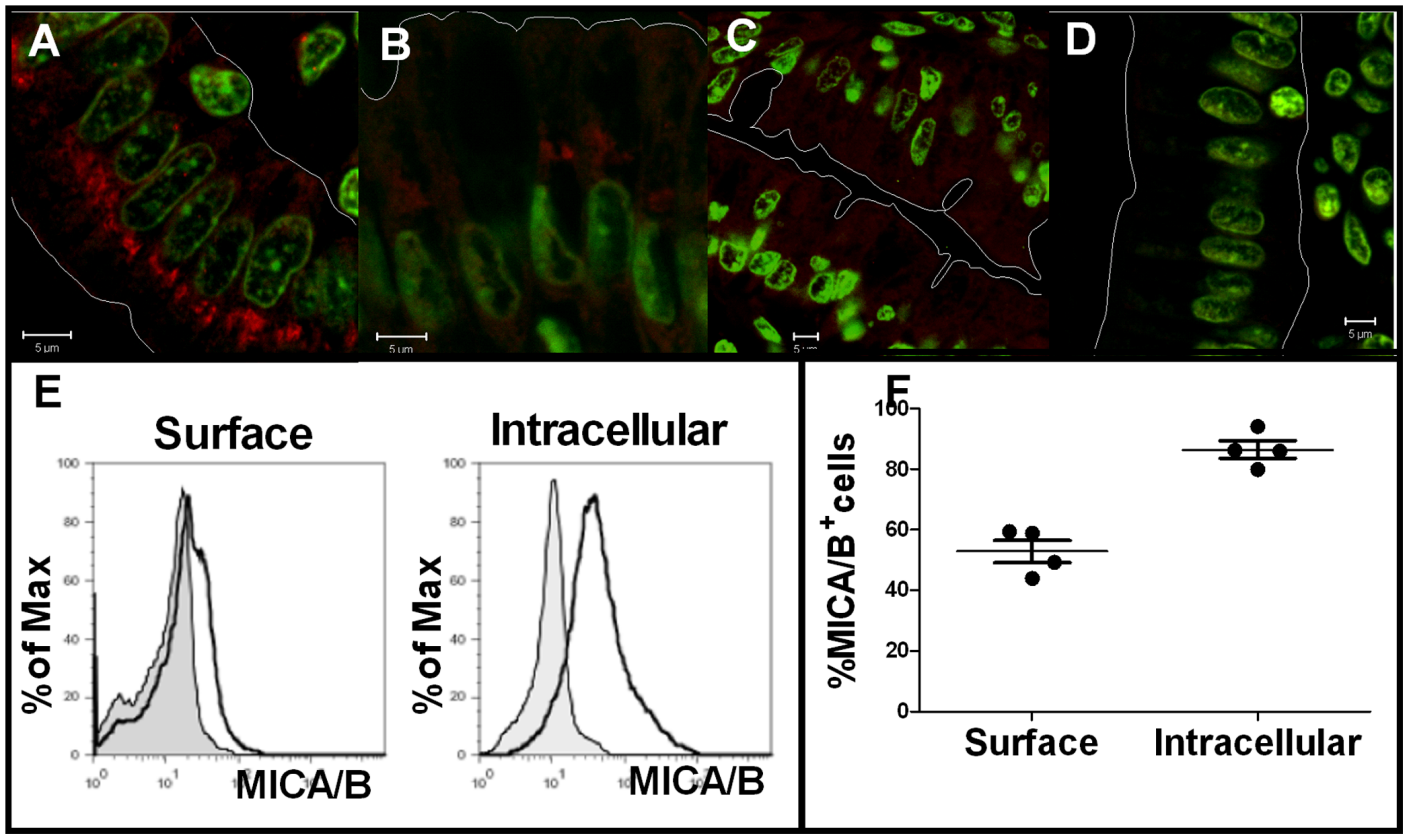
Confocal immunofluorescent analysis showing MICA/B staining. **A.**- sample from an untreated CD pediatric patient with mild enteropathy showing the MICA/B expression (red) in enterocytes. SYTO® 13 (Green Fluorescent Nucleic Acid Stain) was used to stain nuclei. **B.**- MICA/B staining in an intestinal section from the same patient after two years on a gluten-free diet. **C.**- healthy non-celiac control patient. **D.** IC incubated in a section corresponding to sample A. (Magnification 63×). **E.**- Flow cytometric analysis for surface and intracellular expression of MICA/B in epithelial CD3^−^ cells of a representative paediatric patient. **F.**- Flow cytometric analysis for surface and intracellular expression of MICA/B in epithelial CD3^−^ cells of duodenal samples from adult CD patients.

To further investigate the expression of MICA/B in enterocytes, we performed immunofluorescent confocal microscopic analysis. Expression of MICA/B was observed mainly as intracellular staining in enterocytes. In moderate enteropathy, enterocytes showed large MICA/B^+^ aggregates oriented to the apical pole and also associated to the perinuclear region **(** **Figure 2A** **)**. The same pattern was observed in mucosal samples with mild and severe enteropathy (*not shown*). These aggregates were found reduced but did not disappear after at least two years on a gluten-free diet **(** **Figure 2B** **)**. Though samples from non-celiac individuals also showed MICA/B expression in the cytoplasm of enterocytes, the intensity of staining was very low with a diffuse pattern and absence of large aggregates **(** **Figure 2C** **).** Flow cytometric studies on CD3^−^ cells from paediatric duodenal epithelia showed a substantial intracellular expression of MICA/B **(** **Figure 2E** **)**. Similarly, majority of CD3^−^ epithelial cells from duodenal samples of adults CD patients were positive for the intracellular staining, while only half of them were positive for surface MICA/B staining **(** **Figure 2F** **)**.

### Intraepithelial Lymphocytes Express MICA/B

**Figure 3:**
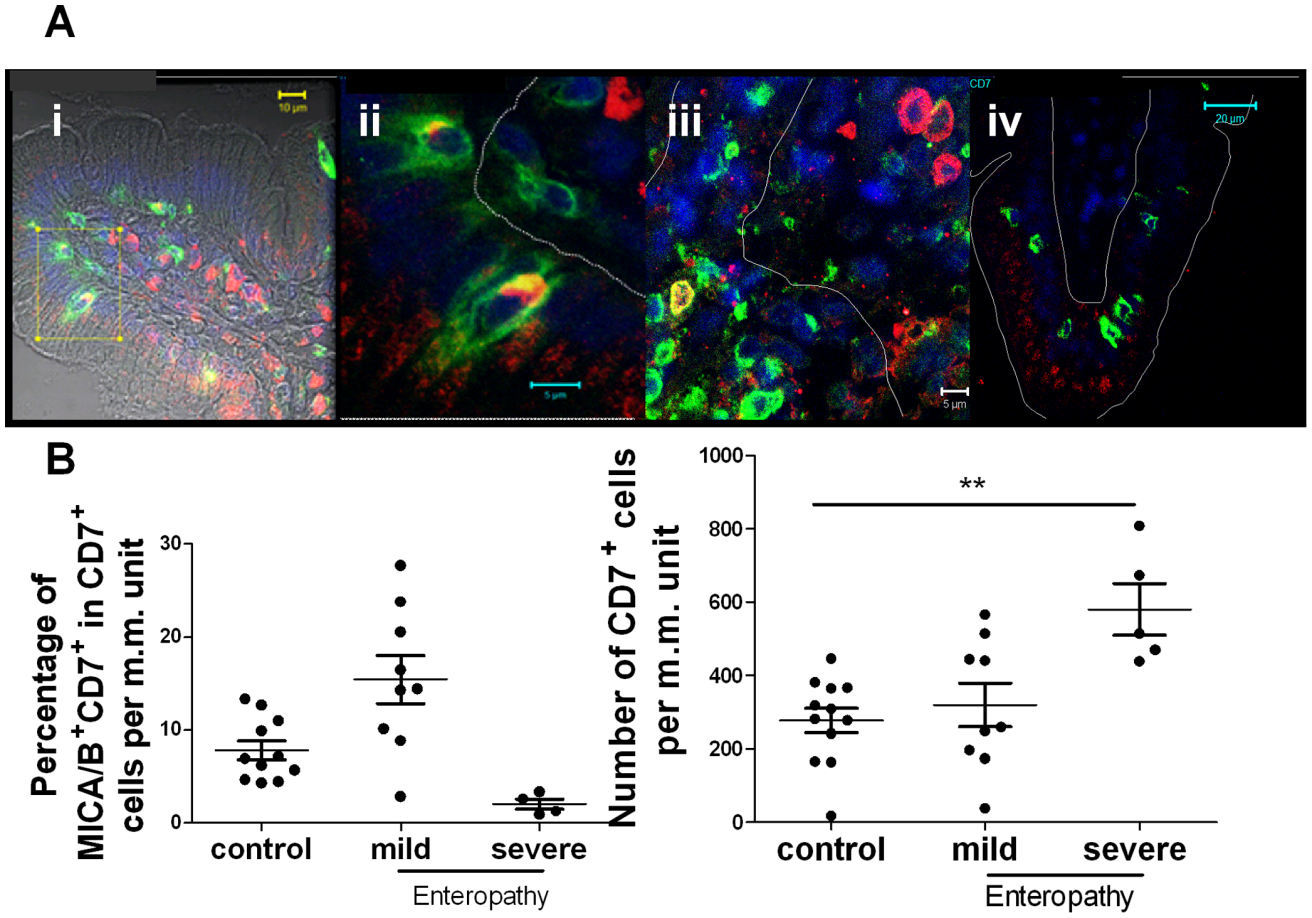
MICA/B^+^ cells in the intraepithelial compartment. **A.**- Immunofluorescent confocal microscopic analysis on small intestinal sections showing CD7^+^ cells (green), MICA/B^+^ cells (red) and nuclei (blue). (i) Mild enteropathy sample (ii) Enlarged section of (i). (iii) Severe enteropathy sample. (iv) Duodenal section from a healthy control. Intraepithelial and *lamina propria* compartments were delimited in the picture with a thin line (scan zoom 0.7, magnification 100×). **B.**- Numbers of CD7^+^MICA/B^+^ were determined per unit of muscularis mucosae m.m. using immunofluorescent microscopy on duodenal sections of 11 healthy controls, 9 patients with mild enteropathy and 4 patients with severe enteropathy. Percentage of CD7^+^MICA^+^ cells (left plot) and total number of CD7^+^ cells (right plot) were depicted. ** p≤0.01, (Non parametric Kruskal wallis test followed by the Dunns multiple-comparison posttest).

As MICA/B expression was also observed in cells in the intraepithelial and lamina propria compartments of small intestine, we performed immunofluorescent confocal microscopy analysis using lineage markers to characterise MICA/B expression in these cells (**Figure 3**). In the intraepithelial compartment, the majority of MICA/B^+^ cells were CD7^+^ cells, confirming that they are intraepithelial lymphocytes (IELs). Particularly, these CD7^+^MICA/B^+^ IELs were abundant in biopsies from patients with mild enteropathy. We also observed that MICA/B staining was intracellular and mainly concentrated in a perinuclear region of the cytoplasm (**Figure 3A**).

Unlike the pattern of staining observed in mild enteropathy, non celiac samples showed very low MICA/B expression in IELs. Scattered CD7^+^ cells mostly presented no MICA/B staining (**Figure 3A** **iv**). The highest percentage (15.6%) of MICA^+^CD7^+^ cells per unit of m.m. was observed in biopsies from patients with mild enteropathy and the total number of MICA^+^CD7^+^ cells was 2.3 times higher than in control samples **(** **Figure 3B** **)**. Surprisingly, in biopsies from patients with severe enteropathy we found the lowest percentages of CD7^+^MICA/B^+^ (2%). In addition, the total number of MICA/B^+^CD7^+^ cells in mild enteropathy was 4.2 times higher than in severe enteropathy, and among IELs, the number of CD7^+^ cells was twice higher in atrophy than that observed in mild enteropathy or control samples. These findings could be associated to the increase in the IEL number characteristically observed in untreated CD.

### Characterisation of MICA/B^+^ Cells in the Small Intestine Lamina Propria

**Figure 4:**
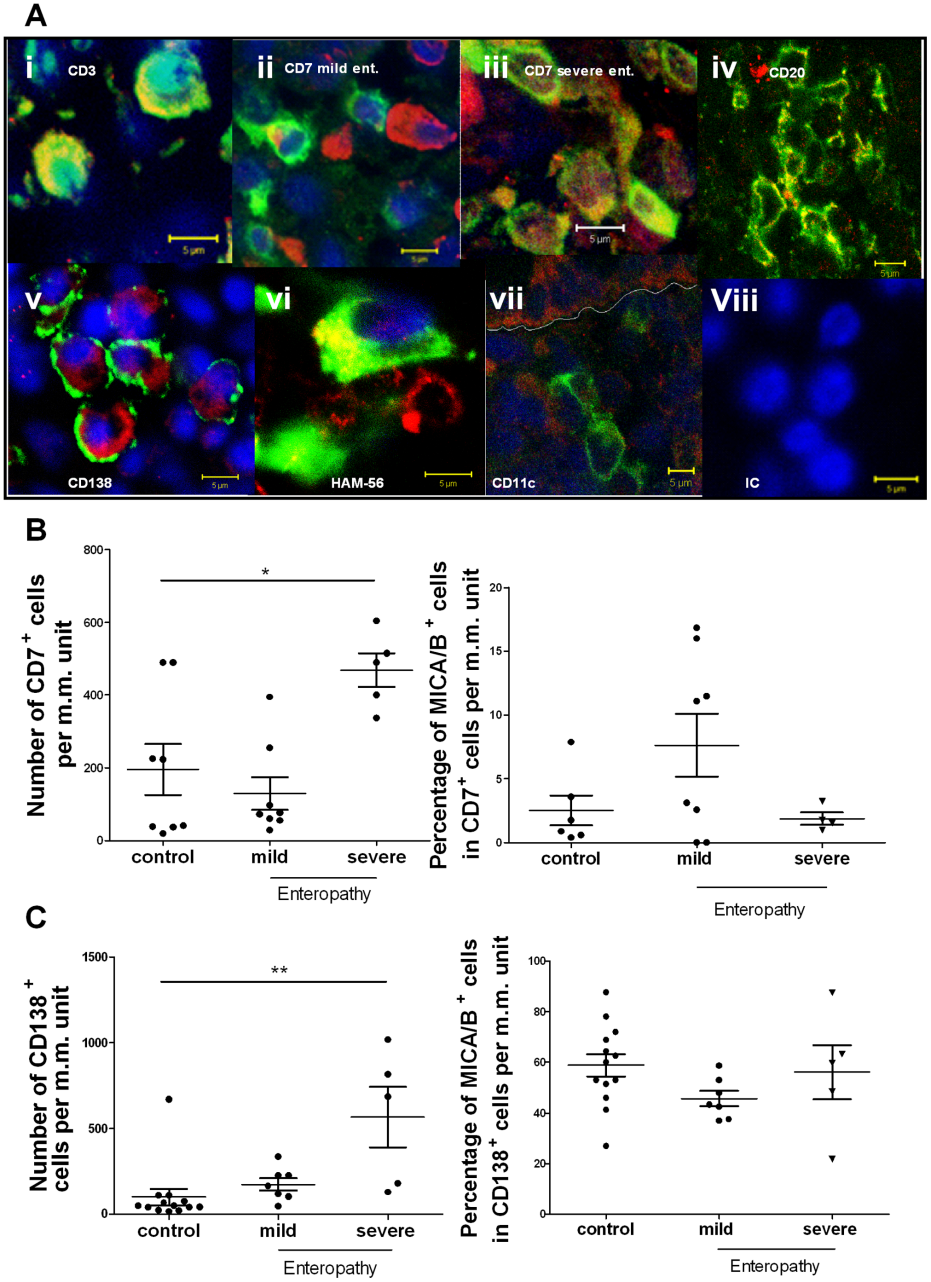
MICA/B^+^ cells in the *lamina propria.* **A.**- Immunofluorescent confocal microscopic analysis was performed in paraffin embedded sections from tissues with severe enteropathy (i, iii, iv, v, vi, vii, viii) and mild enteropathy (ii). Sections were stained as follows: MICA/B (red), Nuclei (blue). i. CD3^+^ cells (green). ii and iii. CD7^+^ cells (green).. iv. CD20^+^ (green). v. CD138^+^ cells (green). vi. HAM-56^+^ cells (green). vii. CD11c^+^ cells (green). viii. IC antibody (all cell lineage markers in green). (scan zoom 0.7, magnification 100×). **B.**- Expression of MICA/B in CD7^+^ cells in sections of small intestine samples of 6 healthy controls, 8 mild enteropathy samples and 4 severe enteropathy samples. Percentage of MICA/B^+^ cells in the CD7^+^ population (left panel) and total number of *lamina propria* CD7^+^ cells per unit of m.m. (right panel) were plotted. * *p*≤0,05; (Non parametric Kruskal wallis test followed by the Dunns multiple-comparison posttest). **C.**- Expression of MICA/B in CD138^+^ cells in sections of small intestine samples of 13 healthy controls, 7 mild enteropathy and 5 severe enteropathy. Percentage of MICA/B^+^ cells on the CD138^+^ population (left panel) and total number of *lamina propria* CD138^+^ cells per unit of m.m (right panel). ** p≤0.01, (Non parametric Kruskal wallis test followed by the Dunns multiple-comparison posttest).

Lymphocytic infiltration in the intestinal mucosa is one of the hallmarks of untreated CD patients. Particularly, the number of *lamina propria* CD3^+^ cells was found dramatically increased in tissues with mild and severe enteropathy, and some of these cells were also MICA/B^+^ (**Figure 4A** **i**). CD7^+^ cells were found isolated or in small groups along the intestinal *lamina propria* of celiacs and non-celiac individuals. Some of these CD7^+^ cells expressed MICA/B (**Figure 4A** **ii and iii)**. Among CD7^+^ *lamina propria* cells, MICA/B^+^ cells represented the 2.6% in controls, the 1.9% in severe enteropathy and the 7.6% in mild enteropathy (**Figure 4B**). Although there were not statistical differences between control and pathological samples, the mean percentage of MICA/B^+^CD7^+^ was higher in mild enteropathy samples. In severe enteropathy, total number of CD7^+^ cells was significantly increased compared to samples from healthy controls, and a twofold increment in the number of MICA/B^+^ *lamina propria* lymphocytes in untreated CD patients compared to healthy controls was found. This finding could be a consequence of the increase in mucosal cellularity in severe enteropathy.

MICA/B expression was also observed in *lamina propria* B cells. CD20^+^ cells were found scattered in the tissue in untreated CD and control samples. Particularly, some of these CD20^+^ cells expressed MICA/B^+^ as shown in severe enteropathy (**Figure 4A** **iv**). In most of the cases, MICA/B staining collocated with the surface lineage B cell marker CD20. To further characterise the expression of MICA/B in the B cell population, we also used the plasma cell marker CD138 (**Figure 4A** **v)**. In severe and mild enteropathy, several aggregates of CD138^+^ cells were found infiltrating the *lamina propria* around the crypts and in the villi. Unlike the pattern observed in CD20^+^ lymphocytes, MICA/B was highly expressed in the cytoplasm of CD138^+^ cells as a perinuclear homogeneous and diffuse ring, and surface CD138 did not collocate with MICA/B. There were no differences in the percentages of CD138^+^ cells among severe or mild enteropathy and control samples. However, total number of CD138^+^ cells was five times higher in severe enteropathy compared to controls. Consequently, the total number of CD138^+^MICA/B^+^ cells in this group was higher compared to controls. This higher number of *lamina propria* plasma cells expressing MICA/B is likely due to the massive increment in cellularity, characteristic of severe enteropathy observed in untreated CD patients **(** **Figure 4C** **)**.

We also assessed the expression of MICA/B in intestinal macrophage/dendritic cell compartment using the following markers: HAM56, CD68, CD1a and CD11c. In only a few cases of severe enteropathy, macrophages HAM56^+^ cells **(** **Figure 4A** **vi)** or CD68^+^ cells (not shown) exhibited MICA/B expression. Moreover, we did not observe expression of MICA/B in CD11c^+^ cells **(** **Figure 4A** **vii)** or CD1a^+^ cells (not shown).

Altogether, these studies demonstrate a broad pattern of expression of MICA/B in cells from the duodenal mucosa. Particularly, we characterised the MICA expression in enterocytes as well as in T lymphocytes (CD7^+^ cells), B lymphocytes (CD20^+^ cells) and plasma cells (CD138^+^) and macrophages (HAM56^+^, CD68^+^ cells).

### Stress Inducers and MICA Expression

**Figure 5:**
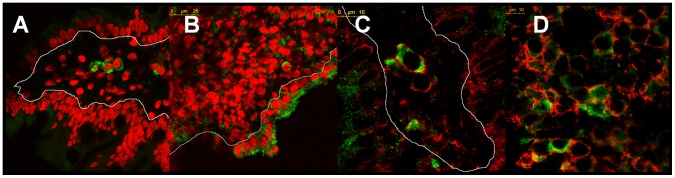
BiP expression in duodenal mucosa. Immunofluorescent confocal analysis on duodenal biopsy samples of a healthy control (A) and a severe enteropathy of a CD patient (B) showing BiP expression (green) and nuclei (red, propidium iodide) (scan zoom 1,7, magnification 63×). Healthy control (C) and severe enteropathy of a CD patient (D) showing BiP (green) and CD138 (red) expression. (scan zoom 4.2 and 3.5, respectively, magnification 63×).

MICA/B was up-regulated by different stress stimuli such as heat shock and oxidants [1]–[6]. The damaged intestinal mucosa is an environment where different stressors may induce such expression. To evaluate whether intestinal tissue shows signs of biological stress, expression of the molecular chaperone BiP or Grp78 (Glucose Regulate Protein 78), a heat shock protein 70 kDa family member known as the master negative regulator of the unfolded protein response (UPR) in mammals [21], was assessed in duodenal biopsies from controls and CD patients. BiP was expressed in *lamina propria* cells in control as well as in CD samples. Remarkably, BiP was strongly upregulated in enterocytes from mucosal tissue of untreated CD patients but not in healthy control samples (**Figure 5A and B**). Some of the *lamina propria* BiP^+^ cells were plasma cells as they stained with anti-CD138 antibodies (**Figure 5C and D**).

**Figure 6:**
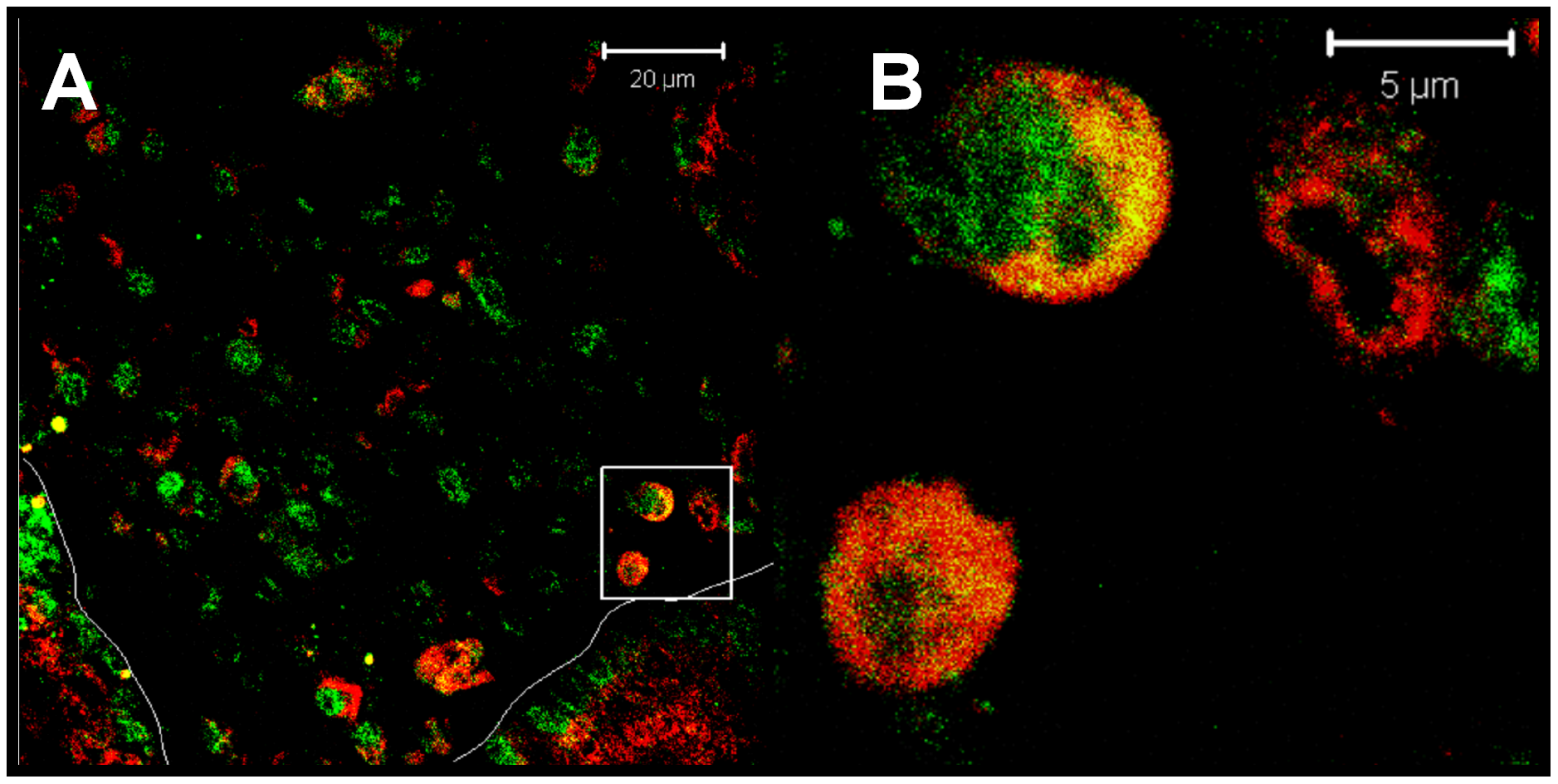
TIA-1^+^ granules indicate stress in the intestinal mucosa in active CD. MICA/B cytoplasmic expression colocalized with TIA-1^+^ granules. (A) MICA/B (red) and TIA-1 (green) in different cell populations in a severe enteropathy of a CD patient. Epithelium was delimited in the picture with a thin line (scan zoom 0.7, magnification 100×). (B) Enlarged picture of (A).

As these findings suggest that stress response is operating at the damaged intestinal mucosa, we next evaluated the expression of TIA-1 (T-cell intracellular antigen), which under stress conditions translocates from the nucleus to the cytoplasm where it appears as part of small cytoplasmic aggregates, known as stress granules [22]. Using immunofluorescent confocal microscopic analysis, we observed co-localization of MICA/B with TIA-1 in the cytoplasm of mononuclear cells in duodenal mucosa in active CD (**Figure 6**), suggesting the existence of an ongoing stress response in CD enteropathy.

**Figure 7:**
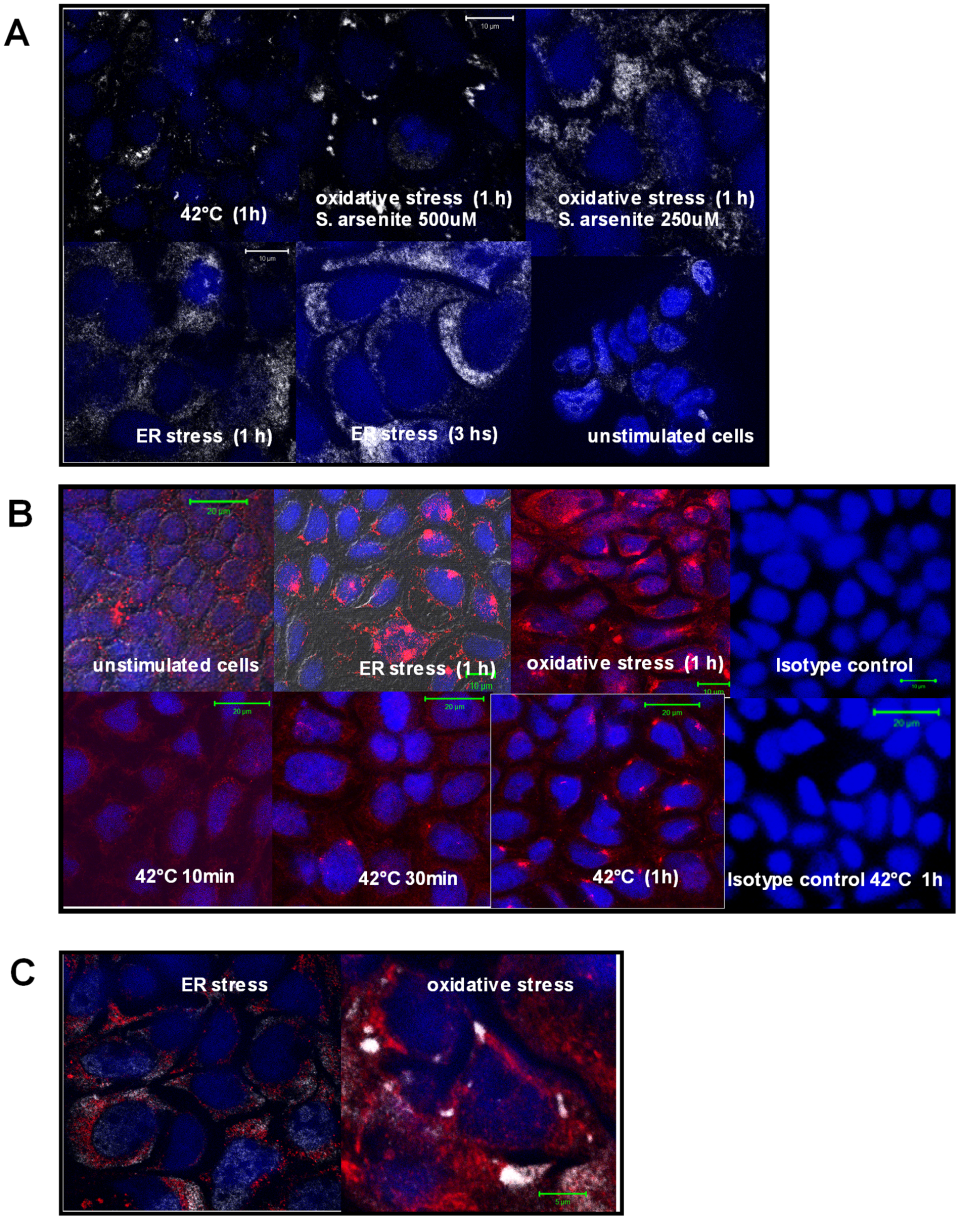
*In vitro* stress treatments change the pattern of MICA/B expression. A. Induction of TIA-1^+^ granules in Caco-2 cells. Confocal microscopic analysis of Caco-2 cells treated during different periods of time with thapsigargin, sodium arsenite or fever-range temperature showing redistribution of TIA-1 (white) into stress granules. Nuclei (blue). (scan zoom 0,7, magnification 100×). **B.**- **Redistribution of MICA/B in treated Caco-2 cells**. Confocal microscopic analysis of Caco-2 cells treated during different periods of time with thapsigargin, sodium arsenite or fever-range temperature showing redistribution of MICA/B (red) in cytoplasmic aggregates. Nuclei (blue). (scan zoom 0,7, magnification 100×). **C.**- **Distribution of MICA/B and TIA-1 in Caco-2 treated cells.** Confocal microscopy of Caco-2 cells treated with thapsigargin (ER stress) or sodium arsenite (oxidative stress) for 1 hour, showing MICA/B (red) and TIA-1 (white) (magnification 100×). In both cases, MICA/B^+^ structures were not associated to stress TIA-1^+^ granules. (scan zoom 0,7, magnification 100×).

We then hypothesized that MICA/B expression is associated to the ongoing stress response in the damaged intestine. To assess whether stress stimuli modulate MICA/B expression, an *in vitro* model consisting of Caco-2 cells was used. Cells were treated with distinct stressors such as thapsigargin (irreversible inhibitor of the sarco-endoplasmic reticulum calcium ATPase –SERCA-, that induces ER stress due to calcium deprivation), sodium arsenite (an oxidative agent) [22] and heat shock (42°C for 1 h) [23], [24]. Thereafter, cellular localization of TIA-1 was analyzed by immunofluorescent confocal microscopy. Incubation of Caco-2 cells under heat shock conditions induced the formation of TIA-1 aggregates compatible with cytoplasmic stress granules (**Figure 7A**). Under oxidative stress conditions we observed that different sodium arsenite concentrations generated different kinds of cytoplasmic TIA-1 aggregates. While one hour incubation with 500 µM sodium arsenite produced the characteristic stress granules TIA-1^+^ in the cytoplasm, fine cytoplasmic TIA-1^+^ aggregates were observed when cells were treated with 250 µM sodium arsenite. We also observed translocation of TIA-1 to the cytoplasm, and the formation of stress TIA-1^+^ granules in cells treated with thapsigargine for one or three hours. In this case, TIA-1^+^ distribution was found as fine cytoplasmic protein aggregates similar to those observed upon exposure of cells to sodium arsenite. These results indicate that all stressors generated a stress response accompanied by TIA-1 translocation to the cytoplasm and stress granules formation in Caco-2 cells.

A kinetic study exposing cells to heat shock during different periods of time showed redistribution of MICA/B into cytoplasmic granules. Analysis by confocal microscopy showed that this redistribution required a treatment longer than 10 min. After 30 min at 42°C, cytoplasmic MICA/B^+^ coarse granules were evident in Caco-2 cells. After one hour of heat shock exposure, most of the cells showed MICA/B^+^ granules. Similarly, these granules were also observed in cells treated for one hour under oxidative or ER stress conditions. Untreated cells showed a diffuse cytoplasmic pattern of MICA/B staining **(** **Figure 7B** **)**.

Cytoplasmic MICA/B^+^ granules formed under oxidative stress did not co-localize with TIA-1^+^ granules; similar results were observed after stress induction with Thapsigargin (**Figure 7C**) and heat shock exposure (not shown).

Altogether, and using a model of human enterocytes (Caco-2 cells), our results suggest that as part of the stress response, MICA/B relocates into cytoplasmic aggregates, and is not *de novo* synthesized. This cytoplasmic location was probed not to be TIA-1^+^ stress granules. In addition, the three *in vitro* models used revealed that under stress conditions MICA/B is redistributed in peri- and/or supra-nuclear coarse granule structures similar to those observed in duodenal mucosa of untreated CD patients and, to a lesser extent, in patients on a gluten free diet.

## Discussion

Celiac disease is characterized by damage to the small intestinal mucosa including villus shortening, crypt hyperplasia, and increased lymphocyte infiltration of the epithelium and *lamina propria* due to an exacerbated proinflammatory immune response to gluten proteins [12]. Several changes are observed in the epithelium, including altered enterocyte shape and height, loss of brush border, vacuolation, denudation and loss of epithelia, some of which are the consequence of increased enterocyte apoptosis [25].

High production of IL-15 in intestinal mucosa in active CD has been shown to trigger enterocyte apoptosis via the induction of cell surface MICA, which in turn interacts with the activating NKG2D receptor present in IELs. Cytotoxic activity of IELs is also potentiated by IL-15 through activation of JNK and ERK pathways [10], [11], [16]. Though MICA/B confers susceptibility to NKG2D-mediated killing of enterocytes by intraepithelial NK and CD8^+^ T cells in untreated CD, our results suggest that MICA/B expression may also regulate cell survival of other cells in the intestinal mucosa.

In our study, we observed a more ubiquitous distribution of MICA/B expression. In enterocytes, the expression was mainly found in the cytoplasm as peri- and/or supra-nuclear aggregates. The analysis of the intraepithelial compartment, which contains different lymphocytes, most of them CD7^+^ cells, revealed the expression of MICA/B in lymphocytes in celiacs and control samples. We found coarse MICA/B aggregates in the cytoplasma of CD7^+^ cells; which were more frequently observed in mild enteropathy samples.

Distinct MICA/B^+^ cell populations such as CD3^+^ and CD7^+^ T lymphocytes, CD20^+^ B lymphocytes and plasma cells were found in the *lamina propria* of non inflammed and enteropathy tissue, and the pattern of the MICA/B staining found in CD7^+^ *lamina propria* cells was coincident with that observed in cells of the intraepithelial compartment. On the other hand, B cells showed clear membrane staining while plasma cells showed an intense but diffuse intracellular pattern. A few HAM 56^+^ macrophages also harbour MICA/B in coarse cytoplasmic aggregates.

MICA/B expression was reduced in duodenal samples from patients under a gluten-free diet, reflecting a possible link between the ongoing inflammatory process induced by gluten ingestion and MICA/B expression. Therefore, considering the pattern of MICA/B expression in different cell lineages observed, signals for induction of MICA/B may be part of a more general mechanism associated to the ongoing inflammatory process in the small intestine in untreated CD patients. Several studies on intestinal tissue, isolated cells from intestinal mucosa or epithelial cell lines support a link between cellular (heat, oxidative and ER) stress and mucosal damage [14], [26]–[29]. Particularly, the occurrence of oxidative stress was observed in intestinal biopsies from untreated CD patients, which was evidenced as increased level of prostaglandins E2 while the levels of the antioxidants enzyme glutathione peroxidase and reductase, and consequently reduced glutathione (GSH), were decreased [30]. In addition, inducible-nitric oxide synthase (iNOS), which is constitutively expressed in duodenal enterocytes, showed increased activity in untreated CD [31]. Direct participation of gliadin peptides, particularly p31–43, in the production of reactive oxygen and nitrogen species (ROS and RNS) has been documented in the induction of oxidative stress in the mucosa of these patients [14]. Therefore, the existence of an altered epithelium as consequence of the oxidative and ER stress might be part of the mechanisms that contribute to the intestinal damage in untreated CD.

To evaluate whether different forms of cellular stress may occur in duodenal mucosa, we analyzed the expression of BiP, a well-established marker of ER stress [21]. BiP was detected in distinct *lamina propria* cells, both in non inflammed tissue and enteropathy. Remarkably, we observed a higher expression of BiP in the epithelia of untreated CD duodenal samples but not in healthy tissue. Therefore, and in accordance with previous studies, our results suggest that an oxidative and an ER stress are present in CD enteropathy [14], [27], [30], [32]. Furthermore, the observation of TIA-1^+^ granules in *lamina propria* mononuclear cells from untreated CD patients further supports this idea.

Immunofluorescent analyses revealed that different cells exhibit a particular pattern of MICA/B staining. These distinct patterns of cytoplasmic MICA/B^+^ structures might be linked to structures formed during the stress response. The RNA binding protein TIA-1, found in small cytoplasmic aggregates, named stress granules [33], was observed in *lamina propria* mononuclear cells, which additionally indicates the existence of an ongoing stress response in duodenal mucosa of untreated CD patients.

*In vitro* studies with Caco-2 cells and different models for oxidative, thermal and ER stress, indicated an accumulation of MICA/B but not in association with TIA-1^+^ stress granules. Although we cannot rule out other intracellular localizations of MICA/B such as associated to aggresomes, this pattern resembles the localization of MICA/B observed in intestinal mucosa in active CD.

Gluten peptides, the causative agent of CD in genetically susceptible individuals, particularly p31–43, may also mediate inflammatory processes [14], [34], alter the traffic of the vesicular compartment resulting in increased epidermal growth factor receptor (EFGR) and the IL-15/IL-15Rα complex expression and activation [35], that altogether contribute to disregulation of tissue remodelling and mucosal damage. Remarkably, gliadin peptides may induce cellular stress in the epithelium by different mechanisms as was observed for oxidative [36] and ER-stress [32] in Caco-2 cells. Enhanced expression of the stress protein HSP65 in epithelial cells in intestine of untreated CD patients appears as result of the chronic inflammation [37]. Altogether, there is substantial evidence indicating that stressed mucosa is a consequence of the inflammatory cascade in CD pathogenesis.

In our study, we also observed expression of MICA/B in B and T lymphocytes. Expression of MICA in activated T lymphocytes has already been observed [38], [39] and it has been reported that such expression confers susceptibility to NK cell-mediated cytotoxicity [40]. More recently, a pathophysiological role of MICA expression and release on T cells during HIV infection was described [41]. Remarkably, we found MICA/B in the cytoplasm of intraepithelial and lamina propria T lymphocytes. Therefore, to the best of our knowledge our findings constitute the first description of *in vivo* expression of MICA in T cells in a pathological situation of non infectious origin such as CD. Expression of MICA/B by T cells makes them susceptible to NKG2D-mediated cytotoxicity by NK cells [40], [42]. Cell surface MICA/B expression may act to negatively regulate T cell function by decreasing of IFN-γ production and cytotoxicity and reduce tissue damage by regulatory mechanisms via NK/T cell interaction. However, intracellular (cytoplasmic) expression, as observed in our study, may preclude that such putative homeostatic mechanism may operate normally and consequently contribute to the tissue damage observed in the mucosa of CD patients [10], [11], [43].

Altogether, our results indicate that the MICA/B expression in intestinal mucosa of celiac patients is indeed broader than originally reported and might be associated to the extensive stress conditions present in the intestinal lesion in active CD. Also, the intracellular location of MICA in intraepithelial and *lamina propria* T cells may hinder their recognition by NKG2D-expressing cells avoiding the control of overactivated T cells, hypothesis to be further investigated in future studies. Therefore, our results suggest that MICA/B may play a more general role than previously thought in gut immunobiology.
